# Supplementary material for: Medical and health students’ insights into mycetoma: A survey-based study on knowledge and clinical practices
Source: PLoS Negl Trop Dis. 2025 Oct 9;19(10):e0013583. doi: 10.1371/journal.pntd.0013583 (PMC12510600; doi:10.1371/journal.pntd.0013583)
Supplement: Attachment — Submitted filename: PLOS MYCETOMA.pdf [file pntd.0013583.s003.pdf]

Author reply

Abstract

The abstract is very long and should be shortened.

Author Reply: Done

In paragraph one of the abstract, these phrases can be removed: 1. a neglected tropical disease endemic in Sudan, 2. recognizing the importance of these competencies, 3. the future frontline health workers.

Author Reply: Done

The abstract does not provide sufficient information about the study's methods. Include a sentence about the tool/questionnaire used for the study.

The abstract should be rewritten to highlight only the most important findings of the study. Start with a background that has one or two sentences stating the aim of the study and/or why it is important. Summarise the methods into two or three sentences. State the study design, number of participants, the period the study was conducted, and the tool used for the study. The next paragraph should summarise the major results/findings of the study. Just start the results without explaining. Sentences like the ones from lines 41 to 43, and 44-46 are not necessary. Have a final paragraph that concludes the abstract. State the overall finding of the study (line 57-59 looks okay for this) and provide recommendations in one or two sentences based on the main findings.

Author Reply: The abstract was re written and structured.

Lines 62-73 should be deleted from the abstract. This can be included in the conclusion of the main manuscript.

Author reply: Okay

Introduction

The introduction is nicely written, but could you add information about how mycetoma spread or is acquired to the first paragraph?

Author reply: Added, lines 153- 156

Materials and methods

Line 190: Explain how the sample size was calculated. Provide the formula and the assumptions made in calculating the sample size.

Author reply: Added

In line 186, you mentioned that universities were randomly selected, and then in line 194 they were selected based on contact availability (suggesting that they were conveniently selected). Can you clarify this?

Author reply:

Thank you for pointing out this inconsistency. To clarify, our original intention was to implement random selection within the stratified public/private categories. However, due to the ongoing conflict in Sudan, the final selection of universities was influenced by the availability and accessibility of contacts, which meant that the process incorporated elements of convenience sampling. We have revised the text to accurately reflect this mixed approach, noting that while stratification was applied to ensure diversity, the constraints of the conflict required adjustments to the sampling strategy.

Divide the first few paragraphs of the methods into these sections: Study design, 2. Study participants (sample size calculations, inclusion and exclusion criteria, sampling technique), 3. Questionnaire (description of the questionnaire)

The paragraph titled piloting and validation, can be added to the Questionnaire section if it is created.

Author reply: Done

I think lines 220-224 would best be added to the questionnaire section to explain how the answers were scored and finally classified into good or poor knowledge and

practice. You need to explain how you selected the cutoffs for defining low, moderate, and high knowledge, as well as poor, acceptable, and good knowledge.

Author reply: Done

The method does not indicate whether ethical approval was obtained for the study.

Author reply: The ethical approval stated under the declaration section, lines 613-616

Results

Remove “I don’t know” from the options for Have you heard of mycetoma.

Author reply: Done

Change Nurse to “Nursing” on table 1

Author reply: Done

Kindly provide a table description of tables 2 and 3 that highlights some of the responses.

Author reply: Done

Is there a reason why the full faculties are presented in Table 1, but in the subsequent tables, only medicine and pharmacy are specified, with the remaining fields grouped into one?

Author reply:

Medicine and pharmacy were the predominant faculties among the study participants, representing the largest proportion of the sample. Therefore, they were presented separately in the subsequent tables to allow for more detailed comparison, while participants from the remaining health-related faculties were grouped together due to their smaller numbers.

I am somewhat concerned about the questions used to assess both mycetoma knowledge and practice. Regarding knowledge assessment, I am wondering how questions K3, K4, K5, and K6 help define whether an individual knows mycetoma. To assess their knowledge, I thought that you would ask them basic questions about the

cause, symptoms, transmission, and treatment/prevention of the disease to see whether they are knowledgeable or not, as you did with the remaining questions.

Author reply:

We appreciate your insightful comment regarding the knowledge assessment questions. The inclusion of items K3, K4, K5, and K6 was intentional, as these questions aimed to evaluate deeper and more specific aspects of mycetoma knowledge, such as understanding of epidemiological trends, risk factors, and disease progression, rather than solely focusing on basic facts like causative agents, symptoms, transmission, and treatment/prevention. While the latter are essential for establishing baseline knowledge, our objective was to capture both fundamental and advanced understanding to better reflect the competence required for effective disease recognition and management. Nevertheless, we acknowledge that the balance between basic and advanced items could be further refined in future studies to enhance construct validity.

I need a little explanation about how the practice assessment items help to define good practice regarding mycetoma. Are the questions aimed at understanding whether participants can perform those activities or to know whether they have done those activities before?

Author reply:

Thank you for your question. The practice assessment items were designed to evaluate whether participants have the necessary skills and competencies to perform key activities related to mycetoma diagnosis, management, and prevention, rather than simply recording whether they had previously carried out these activities. This approach allowed us to assess their potential practical readiness, even if clinical or community-based opportunities to apply these skills had not yet arisen.

I suggest merging tables 4 and 5 to tables 2 and 3.

Author reply:

Thank you for your suggestion. We believe it is better to keep Tables 4 and 5 separate from Tables 2 and 3, as they present distinct analytical outputs. Tables 2 and 3 focus on descriptive findings of knowledge and practice by faculty. Keeping them separate

ensures clarity and helps readers interpret the results without conflating descriptive and inferential data.

Please, provide the table associated with the “ANOVA analysis”. I think that if you want to find out how the demographics of the participants are associated with knowledge and practice regarding mycetoma, then reclassify the scores dichotomously into good and poor knowledge/practice. This way, you can use logistic regression (instead of ANOVA) to determine the factors associated with knowledge/practice.

Author reply:

Thank you for your insightful comment. The ANOVA table was not included in the manuscript to maintain brevity but can be provided as supplementary material if required. We acknowledge that reclassifying the scores into dichotomous categories (good vs. poor knowledge/practice) and applying logistic regression would provide a more robust analysis of the associations between demographic factors and knowledge/practice levels. However, our primary aim was to explore mean differences across groups, which is why ANOVA was initially selected. We will consider incorporating logistic regression in future analyses or in a revised version of the manuscript to strengthen the interpretation of these associations.

Discussion

The authors stated that a substantial portion of the students demonstrated knowledge of mycetoma. But from Table 4, only 26.3% had high knowledge of mycetoma. This makes me wonder how a good overall knowledge was defined. At what percentage is a good knowledge defined?

Author reply:

Thank you for pointing this out. To clarify, our statement referred to a substantial proportion of students demonstrating *familiarity* with mycetoma, not solely high-level knowledge. This familiarity indicates that educational efforts—whether through formal

curricula, social media, or community health initiatives—have begun to raise awareness among future healthcare providers. While only 26.3% achieved a “high knowledge” score based on our predefined cut-off, many others possessed partial or basic knowledge, which still reflects an encouraging level of exposure to the disease in this population.

Line 370 states that 80.6% of the students have heard about mycetoma. Where is that reported in the results? Again, since knowledge was not assessed with a single question but a set of questions, overall knowledge should be defined by a composite value such as the ones found in Table 4.

Author reply:

Thank you for your observation. The figure of 80.6% of students having heard about mycetoma is reported in **Table 1, item number 5** (following “academic year”). We agree that overall knowledge was assessed using a composite score derived from the set of knowledge questions, as presented in Table 4, rather than from a single awareness question.

Please include the limitations of the study to the manuscript.

Author reply:

Included
