# Supplementary material for: Medical and health students’ insights into mycetoma: A survey-based study on knowledge and clinical practices
Source: PLoS Negl Trop Dis. 2025 Oct 9;19(10):e0013583. doi: 10.1371/journal.pntd.0013583 (PMC12510600; doi:10.1371/journal.pntd.0013583)
Supplement: Attachment — Submitted filename: PLOS MYCETOMA.docx [file pntd.0013583.s001.docx]

Thank you for inviting me to review the manuscript for such an important topic. I believe that a lot of work went into the research. Much effort was also put into the writing of the manuscript. However, I have some concerns and suggestions stated below, which I believe could help improve the manuscript for publication.

Abstract

The abstract is very long and should be shortened.

In paragraph one of the abstract, these phrases can be removed: 1. a neglected tropical disease endemic in Sudan, 2. Recognising the importance of these competencies, 3. the future frontline health workers.

The abstract does not provide sufficient information about the study's methods. Include a sentence about the tool/questionnaire used for the study.

The abstract should be rewritten to highlight only the most important findings of the study. Start with a background that has one or two sentences stating the aim of the study and/or why it is important. Summarise the methods into two or three sentences. State the study design, number of participants, the period the study was conducted, and the tool used for the study. The next paragraph should summarise the major results/findings of the study. Just start the results without explaining. Sentences like the ones from lines 41 to 43, and 44-46 are not necessary. Have a final paragraph that concludes the abstract. State the overall finding of the study (line 57-59 looks okay for this) and provide recommendations in one or two sentences based on the main findings.

Lines 62-73 should be deleted from the abstract. This can be included in the conclusion of the main manuscript.

Introduction

The introduction is nicely written, but could you add information about how mycetoma spread or is acquired to the first paragraph?

Materials and methods

Line 190: Explain how the sample size was calculated. Provide the formula and the assumptions made in calculating the sample size.

In line 186, you mentioned that universities were randomly selected, and then in line 194 they were selected based on contact availability (suggesting that they were conveniently selected). Can you clarify this?

Divide the first few paragraphs of the methods into these sections: Study design, 2. Study participants (sample size calculations, inclusion and exclusion criteria, sampling technique), 3. Questionnaire (description of the questionnaire)

The paragraph titled piloting and validation, can be added to the Questionnaire section if it is created.

I think lines 220-224 would best be added to the questionnaire section to explain how the answers were scored and finally classified into good or poor knowledge and practice. You need to explain how you selected the cutoffs for defining low, moderate, and high knowledge, as well as poor, acceptable, and good knowledge.

The methods does not indicate whether ethical approval was obtained for the study.

Results

Remove “I don’t know” from the options for Have you heard of mycetoma.

Change Nurse to “Nursing” on table 1

Kindly provide a table description of tables 2 and 3 that highlights some of the responses.

Is there a reason why the full faculties are presented in Table 1, but in the subsequent tables, only medicine and pharmacy are specified, with the remaining fields grouped into one?

I am somewhat concerned about the questions used to assess both mycetoma knowledge and practice. Regarding knowledge assessment, I am wondering how questions K3, K4, K5, and K6 help define whether an individual knows mycetoma. To assess their knowledge, I thought that you would ask them basic questions about the cause, symptoms, transmission, and treatment/prevention of the disease to see whether they are knowledgeable or not, as you did with the remaining questions.

I need a little explanation about how the practice assessment items help to define good practice regarding mycetoma. Are the questions aimed at understanding whether participants can perform those activities or to know whether they have done those activities before?

I suggest merging tables 4 and 5 to tables 2 and 3.

Please, provide the table associated with the “ANOVA analysis”. I think that if you want to find out how the demographics of the participants are associated with knowledge and practice regarding mycetoma, then reclassify the scores dichotomously into good and poor knowledge/practice. This way, you can use logistic regression (instead of ANOVA) to determine the factors associated with knowledge/practice.

Discussion

The authors stated that a substantial portion of the students demonstrated knowledge of mycetoma. But from Table 4, only 26.3% had high knowledge of mycetoma. This makes me wonder how a good overall knowledge was defined. At what percentage is a good knowledge defined?

Line 370 states that 80.6% of the students have heard about mycetoma. Where is that reported in the results? Again, since knowledge was not assessed with a single question but a set of questions, overall knowledge should be defined by a composite value such as the ones found in Table 4.

Please include the limitations of the study to the manuscript.
